# Metastatic right atrial mass in the presence of atrial septal defect: A rare clinical coincidence

**DOI:** 10.1002/ccr3.8916

**Published:** 2024-06-05

**Authors:** Alireza Arzhangzadeh, Ahmad Ali Amirghofran, Roozbeh Narimani Javid, Vahid Mohammadkarimi, Firoozeh Abtahi, Mohammad Rafati Navaei, Salma Nozhat, Sarvenaz Salahi, Sasan Shafiei, Soorena Khorshidi

**Affiliations:** ^1^ Department of Cardiology, School of Medicine Shiraz University of Medical Sciences Shiraz Iran; ^2^ Department of Surgery Shiraz University of Medical Sciences Shiraz Iran; ^3^ Student Research Committee Hamadan University of Medical Sciences Hamadan Iran; ^4^ Department of Internal Medicine, School of Medicine Shiraz University of Medical Sciences Shiraz Iran; ^5^ Royan Stem Cell and Biotechnology Research Center Tehran Iran; ^6^ Minimally Invasive Surgery Research Center Tehran Iran; ^7^ Department of Cardiology Shiraz University of Medical Sciences Shiraz Iran

**Keywords:** ASD, atrial mass, atrial septal defect, mediastinum mass

## Abstract

**Key Clinical Message:**

The key takeaway from this clinical scenario is to choose the most appropriate and reasonable treatment plan when dealing with a patient who has atrial septal defect (ASD) and concurrent atrial and mediastinal masses. In such cases, a heart‐oncology team should make the therapeutic decision.

**Abstract:**

Right atrial masses are not pretty rare and might be a diagnostic challenge. Thrombosis, tumors, and vegetations are primary differential diagnoses. Workup for these masses usually includes multimodality imaging and biopsy in selected cases. We report a case of a 37‐year‐old lady who presented with cough, dyspnea, and head and neck swelling after a cesarean section. Echocardiography revealed a right atrial mass accompanied by a secundum type atrial septal defect (ASD). Pulmonary CT Angiography was performed, in which a lobulated mass in the anterior mediastinum was detected, and a heart‐oncology team made the therapeutic decision. The patient was scheduled for surgical ASD closure and concomitant tissue biopsy. The pathology results were in favor of poorly differentiated germ cell tumors, and chemotherapy was started following the surgery. After two sessions of chemotherapy, the tumor did not respond to the primary regimen. Thus, an updated regimen was initiated. Compliance with the updated regimen was acceptable, and the patient is currently under treatment and follow‐up.

## 1 INTRODUCTION

Cardiac masses are generally infrequent. Nevertheless, among all the chambers of the heart, the presence of a mass in the right atrium poses distinct difficulties due to typical structural variations that can imitate a tumor. Furthermore, the presence of indwelling catheters and pacemaker leads in the right atrium elevates the likelihood of thrombi or vegetations, which can also mimic a tumor.^1^

The primary cause of a mass in the right atrium is typically a thrombus resulting from the connection between the right atrium and the deep venous system.^2^ However, the presence of a mass in the right atrium should raise concerns about the possibility of a neoplasm, which can be either benign or malignant. Myxoma, the most prevalent noncancerous neoplasm, is usually connected by a stalk in close proximity to the fossa ovalis. Alternatively, malignant cardiac neoplasms may be due to the metastasis or proliferation and expansion of nearby tumors. Primary malignant tumors of the right atrium are uncommon, with a metastatic to primary ratio of 40:1. These tumors include angiosarcoma, lymphoma, and pericardial mesothelioma.^3^

Germ cell tumors (GCTs) or teratomas are a rare type of cardiac neoplasms, with just a few cases reported, particularly in adults. The majority of the documented instances involve secondary metastases originating from the testicles or primarily located in the pericardium. However, these cases are predominantly observed in pediatric patients.^4^

In this article, we describe a clinical scenario of right atrial mass and synchronous secundum type atrial septal defect (ASD) in the presence of a large mediastinal mass, an experience that is rare but well worth sharing for its diagnostic and surgical challenges.

## 2 CASE PRESENTATION

A 37‐year‐old woman was brought to the ED with a recent onset cough, dyspnea, and head and neck swelling after a cesarean section operation 2 weeks ago. Facial congestion and difficulty breathing were noted in pregnancy but worsened in the last few days. She had a nonproductive cough but no hemoptysis. There was no history of cardiac or pulmonary disease or alcohol, tobacco and illicit drugs.

On physical examination, the heart rate was 110 beats per min, and blood pressure was 110/75 mmHg. She was afebrile. There was no cyanosis but slight respiratory discomfort. Auscultation revealed clear lungs. Heart examination was negative for murmur, but wide fixed S2 splitting was noted. There was no evidence of right heart failure. The patient was admitted for further investigations.

## 3 INVESTIGATIONS AND TREATMENT

**FIGURE 1:**
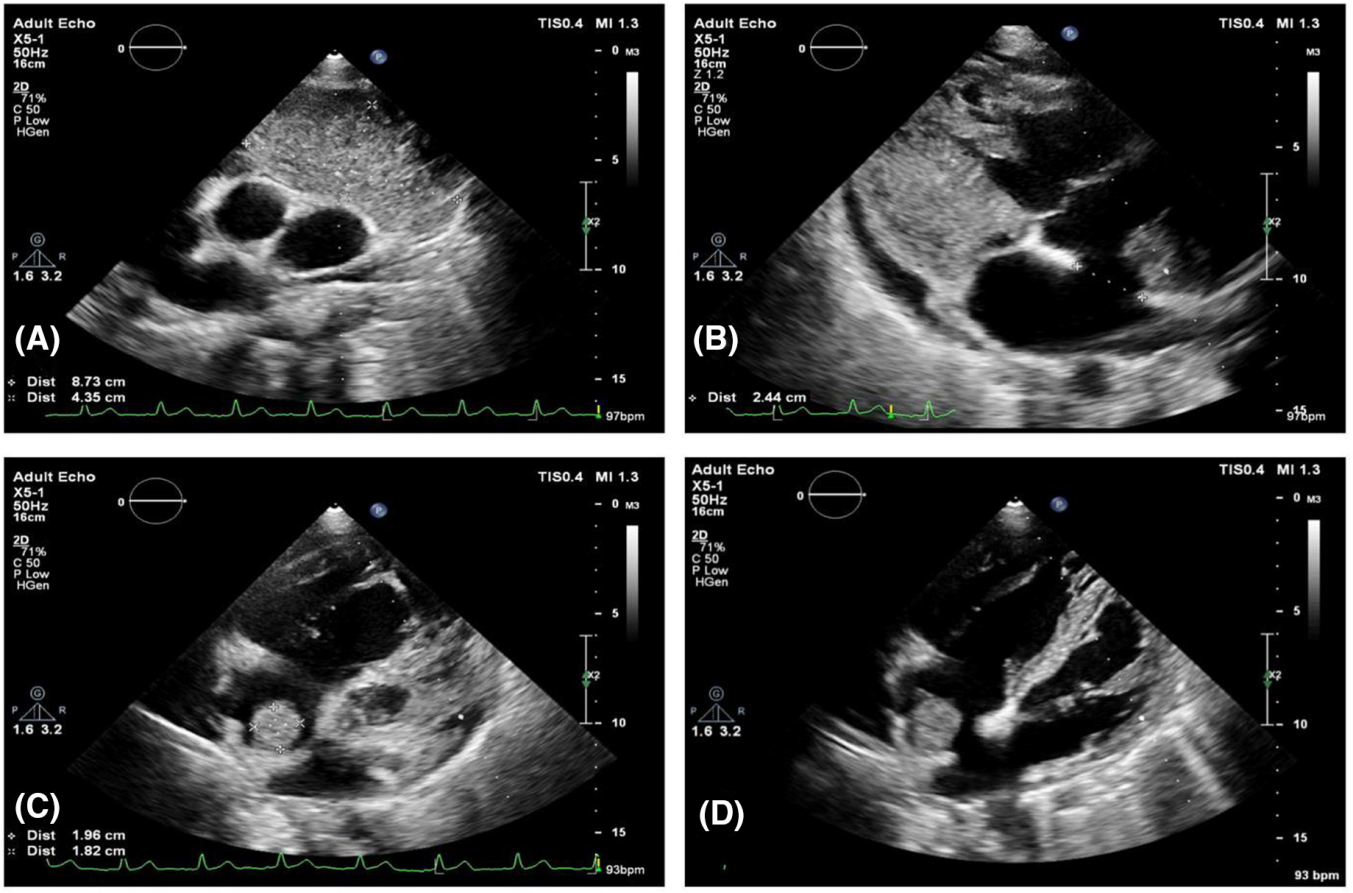
Echocardiography. Evidence of large anterior to ascending aorta (A). Large ASD secundum type, 2.4 cm (B). Large nonmobile mass in RA, attached to RA wall (C, D).

Our patient was planned for echocardiography due to the postpartum status, respiratory distress, and cardiac auscultation findings. The echocardiography demonstrated a severely dilated RA and RV accompanied by a nonmobile mass measuring 6.5 cm^2^ attached to RA wall and secundum type ASD with a diameter of 2.44 cm and a net left to right shunt with QP/QS 2.9 (Figure 1). Besides these findings, a large extracardiac mass anterior to the ascending aorta (8.7 × 4.3 cm) most compatible with malignancy was also noted.

**FIGURE 2:**
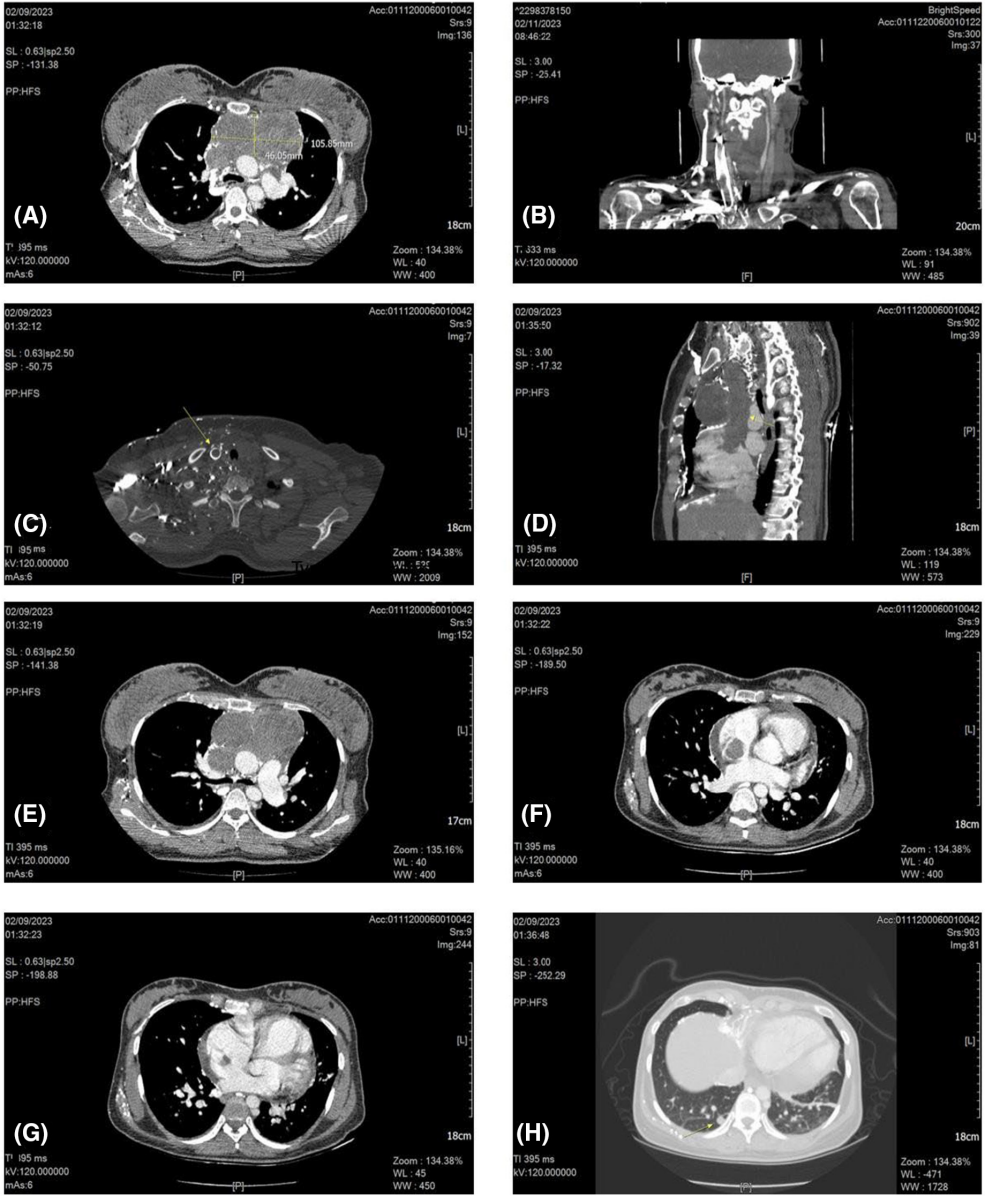
Pulmonary CT angiography. Evidence of a heterogeneous lobulated hypo‐dense mass measuring 105 × 6 mm is seen at the anterior mediastinum (A). Evidence of extension of tumor thrombosis into the right jugular vein is seen (B, C). Evidence of extension of tumor thrombosis into the superior vena cava is seen (D). Evidence of a large heterogeneous hypo‐dense lobulated mass extending to the superior vena cava is seen (E). Extension of tumor thrombosis from the superior vena cava into the right atrium is seen (F). Evidence of ASD is present (G). Evidence of multiple lung nodules up to 10 ×9 mm in the basal segment of the right lower lobe due to lung metastasis is seen in both lung fields (H).

For further evaluation, the patient underwent a thoracic CT scan and Pulmonary CT Angiography (Figure 2), which revealed a lobulated soft tissue mass in the anterior mediastinum measuring 105 × 46 mm, mostly compatible with thymomas, teratomas, thyroid goiters, lymphomas, and GCTs.^5^ In addition, thrombosis of brachiocephalic veins and SVC associated with significant collateral formation in favor of SVC syndrome was also noted. It was reported that the atrial mass was likely an extension of thrombosis or malignant invasion in the RA. CT scan also revealed several nodules in both lung fields, indicating multiple lung metastases. The patient also underwent abdomen and pelvic CT, which showed no abnormal findings or distal metastases. Blood work was remarkable for an AFP level of 5000 ng/mL, and the blood culture was negative.

At this point, by considering the findings of physical and laboratory examinations, echocardiography, thoracic CT scan, and pulmonary CT angiography, a mediastinal malignancy with cardiac and lung metastasis was at the top of our differential diagnoses. Atrial thrombosis was another potential diagnosis due to the hypercoagulable state because of the post‐partum period and the mediastinal probable malignancy. On the other hand, the absence of fever, negative blood culture, and no history of IV drug abuse made endocarditis vegetation a less likely diagnosis.

To address the situation, we gathered a heart‐oncology team and made a decision based on a teamwork approach. Surgical excision of the mediastinal mass was not possible due to the large size of the tumor, embedded vessels, and metastasis. Paradoxical necrotic tissue embolization via ASD following chemotherapy as a part of the primary mediastinal mass treatment was another issue that could not be overlooked. It necessitated ASD closer prior to any other treatment.

ASD device closure was considered a possible choice. However, the major obstacle to this strategy was the proximity of RA mass to ASD and the very high risk of iatrogenic embolization. On the other hand, by conducting the surgical ASD closure, the concurrent excision of the atrial mass and histological evaluations were also feasible. Due to the huge anterior mediastinal mass over the great vessels, the risk of median sternotomy was very high. Thus, after a comprehensive evaluation, the team's collective decision was made, and the patient was scheduled for surgical ASD closure and concomitant tissue biopsy as soon as possible.

To decrease unnecessary dissection and necrotic mass entrance, the right lateral thoracotomy approach was selected. The CPB was initiated after cannulation of the right femoral artery and right femoral and axillary veins with vacuum‐assisted venous drainage. The pericardium was identified and opened. The aorta was clamped, and as the aortic valve was patent without regurgitation, the heart fibrillated, and the right atrium was opened. There was a fragile and necrotic mass over the border of ASD. The left atrium was packed with gauze to reduce the risk of emboli during resection. A bovine patch was used to close the ASD after the atrial mass resection. An aorta decamp was performed after heart de‐airing. Following the chest tube insertion, the chest wall was successfully closed, and the patient was weaned from CPB. It was a smooth post‐op process.

Biopsies of the dissected mass were performed, and slides were reviewed by a pathology specialist. The final result identified GCTs with poorly differentiated characteristics. After the convalescent period of the surgery, chemotherapy with the BEP regimen (bleomycin, etoposide, platinum [cisplatin]) was initiated for our patient.

## 4 OUTCOME AND FOLLOW‐UP

The patient tolerated two sessions of chemotherapy well. However, as AFP increased to 12,000 ng/mL, the multidisciplinary team decided to change the regimen to TIP (paclitaxel, ifosfamide, and cisplatin) due to the possible unresponsiveness of the tumor to the primary chemotherapy regimen. Her compliance with the updated regimen was also acceptable, and she is currently under treatment and follow‐up.

## 5 DISCUSSION

In this clinical scenario, we presented a 37‐year‐old lady with a right atrial mass and synchronous secundum type ASD in the presence of a large mediastinal mass. The atrial mass was found to be the metastasis of a mediastinal germ cell tumor.

GCTs account for about 15%–20% of all anterior mediastinal masses. They are most commonly found in individuals aged 20–40, with an equal distribution between males and females. The majority of GCTs are non‐malignant, with teratoma being the most prevalent kind.^6^ Theoretically, tumor metastasis to the heart can happen through various mechanisms such as direct extension, hematogenous spread, lymphatic drainage, and intracavitary diffusion via the superior vena cava or pulmonary veins.^7^

Elevated levels of serum alpha phetoprotein (AFP), human chorionic gonadotropin (HCG), and lactate dehydrogenase (LDH) may be observed in patients with mixed GCT.^8^ Imaging investigations, such as CT scans, usually reveal a large, heterogeneous mass with necrosis, hemorrhage, and frequent invasion of nearby tissues.^9^

The current standard therapies for individuals with mediastinal mixed GCTs involve chemotherapy followed by surgical removal of persistent mass.^10^ The surgical technique depends mostly on the type of GCT, with the goal of achieving total removal. Complete surgical resection is a crucial determinant of the prognosis of mediastinal GCTs.^8^ In our case, the surgical excision of mediastinal GCT was not feasible due to the huge anterior mediastinal mass over the great vessels and lung and cardiac metastasis.

However, because of the high probability of necrotic tissue embolization of the right atrial mass following chemotherapy, atrial mass and ASD were addressed surgically prior to the chemotherapy. According to the need for concurrent atrial mass excision and ASD closure, the proximity of RA mass to ASD, and the very high risk of iatrogenic embolization, ASD device closure was not also applicable.

**FIGURE 3:**
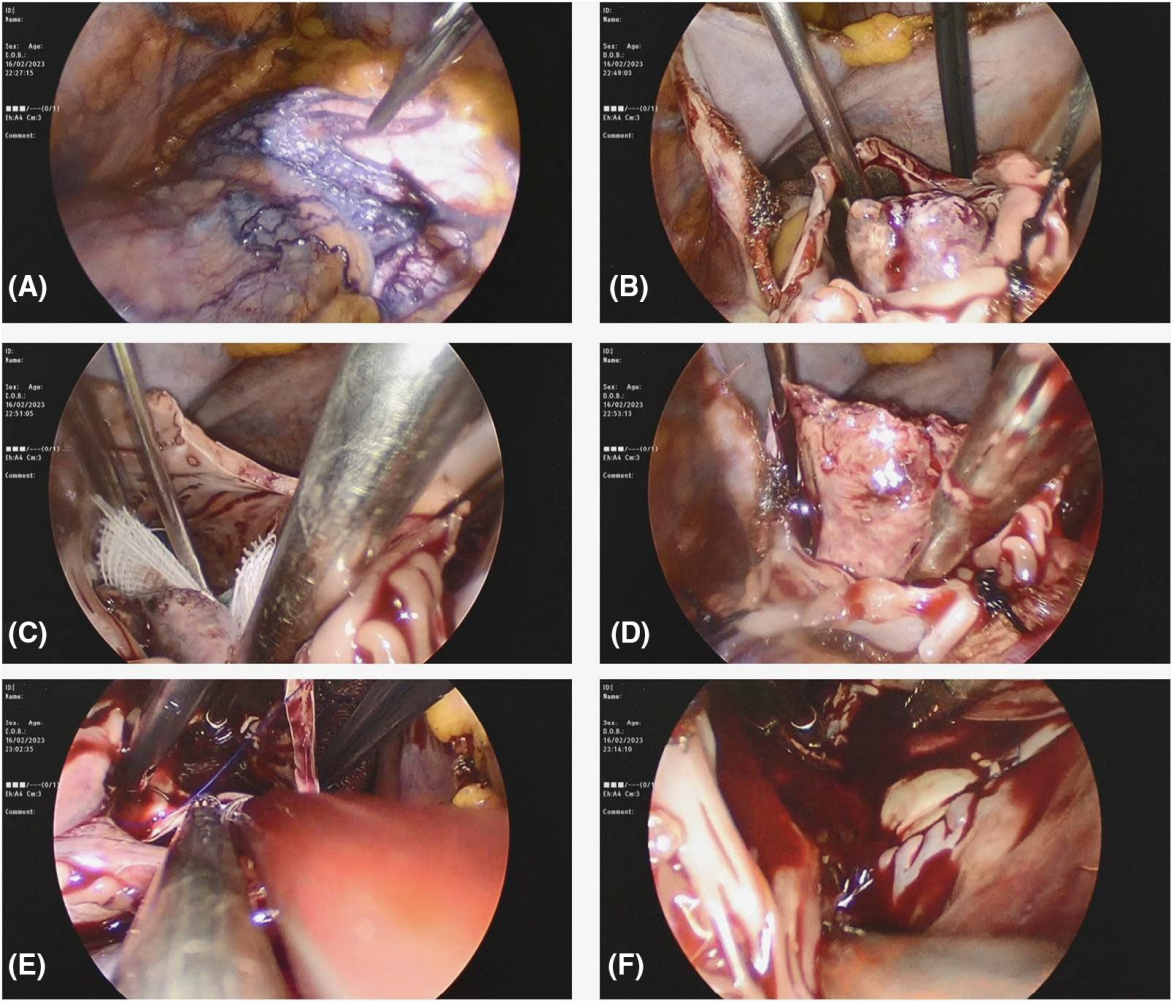
Intraoperative images. High‐density collateral vessels on the epicardial surface of the heart due to SVC obstruction (A). Right atrial mass exposure (B). Applying surgical gauze in ASD to avoid embolization (C). Amputation of necrotic mass from stalk in SVC (D). Minimally invasive ASD closure with bovine patch (E). Final RA view with removed mass and closed ASD (F).

Direct examination of the right atrial mass in the operation room revealed that the mass was an extension of metastasis in SVC (Figure 3). Starting chemotherapy without ASD closure with such kind of a highly necrotic and fragile tumor could lead to catastrophic embolic events. ASD repair prior to chemotherapy was likely high risk but presumably the most efficient.

The presence of a right atrial mass with a synchronous atrial septal defect is a rare clinical situation. There are only a few similar reports. Ozawa reported a case of primary malignant fibrous histiocytoma of the heart with a left‐to‐right atrial shunt. It was presumed that the shunt was caused by the extension of the tumor along the rims of the previous patent foramen oval or that there was a small unrecognized ASD. Their patient died 6 months after the first presentation, and the diagnosis was confirmed with a postmortem autopsy.^11^ Chamsi‐Pasha et al. The report describes a successfully excised right atrial myxoma and ASD closure in a 40‐year‐old woman.^12^ Slovis et al. a patient with pulmonary capillary hemangiomatosis and sinus venosus ASD died due to refractory right‐sided heart failure.^13^

The overall survival (OS) of patients with GCTs is significantly associated with the surgical removal of the tumor. This procedure eliminates tumor tissue that is resistant to chemotherapy and allows for histological examination, which aids in evaluating the response to chemotherapy and planning future treatment strategies.^14^

However, many patients, like ours, may not be able to have surgery due to factors such as greater tumor size, embedment of vessels, and the presence of metastases at the time of diagnosis. The patients are recommended to undergo chemotherapy using the EP regimen (etoposide plus cisplatin) or the paclitaxel plus ifosfamide plus cisplatin regimen.^8^ Administering chemotherapy following the removal of any remaining mass is also crucial in order to effectively manage the morbidity associated with GCT.^15^

The response to chemotherapy can be evaluated via different serological tumor makers, including AFP.^16^ In our case, the primary chemotherapy regimen failed to decrease AFP. However, a favorable response was obtained with an update on the regimen. Ultimately, Mediastinal GCTs typically have unfavorable prognoses, and based on a recent study,^17^ the three‐year OS rate for patients with non‐seminomatous GCT (NSGCT) is only 26%. The 3‐year OS rate of patients with NSGCT who had surgery to remove the remaining mediastinal tumor following receiving chemotherapy is about 59%.

## AUTHOR CONTRIBUTIONS

**Alireza Arzhangzadeh:** Writing – original draft; writing – review and editing. **Ahmad Ali Amirghofran:** Conceptualization; investigation. **Roozbeh Narimani Javid:** Validation; writing – review and editing. **Vahid Mohammadkarimi:** Investigation; writing – review and editing. **Firoozeh Abtahi:** Writing – original draft. **Mohammad Rafati Navaei:** Investigation; writing – original draft. **Salma Nozhat:** Writing – review and editing. **Sarvenaz Salahi:** Conceptualization. **Sasan Shafiei:** Writing – review and editing. **Soorena Khorshidi:** Project administration; writing – review and editing.

## FUNDING INFORMATION

The authors received no specific funding for this work.

## CONFLICT OF INTEREST STATEMENT

The authors declare they have no competing interests.

## ETHICS STATEMENT

Ethics approval was not sought as this report contains a single case report for which patient consent was obtained.

## CONSENT

Written informed consent was obtained from the patient to publish this report in accordance with the journal's patient consent policy.

## Data Availability

The data that support the findings of this study are available on request from the corresponding author, [S.Kh]. The data are not publicly available due to containing information that could compromise the privacy of research participant.
